# Complex Genomic Rearrangement Involving the *TBX4* Promoter Manifesting With Variable Expressivity in a Five‐Generation Family

**DOI:** 10.1155/humu/7278477

**Published:** 2026-08-03

**Authors:** Shruti A. Pande, Hiuling Chan Joiner, Przemyslaw Szafranski, Tomasz Gambin, Frank P. Edenborough, Rachael E. Thompson, Michael J. Parker, Carrie Hammond, Shahin Moledina, Justyna A. Karolak, Pawel Stankiewicz

**Affiliations:** ^1^ Department of Molecular and Human Genetics, Baylor College of Medicine, Houston, Texas, USA, bcm.edu; ^2^ Institute of Computer Science, Warsaw University of Technology, Warsaw, Poland, pw.edu.pl; ^3^ Sheffield Adult Cystic Fibrosis Centre, Sheffield Teaching Hospitals NHS Foundation Trust, Northern General Hospital, Sheffield, UK, nhs.uk; ^4^ Sheffield Clinical Genetics Service, Sheffield Children’s NHS Foundation Trust, Northern General Hospital, Sheffield, UK, nhs.uk; ^5^ Great Ormond Street Hospital for Children NHS Foundation Trust and UCL Institute of Cardiovascular Sciences, London, UK; ^6^ Chair and Department of Genetics and Pharmaceutical Microbiology, Poznan University of Medical Sciences, Poznan, Poland, ump.edu.pl

**Keywords:** incomplete penetrance, lung diseases, noncoding modifiers, small patella syndrome

## Abstract

Variants involving *TBX4* are associated with ischiocoxopodopatellar syndrome with or without pulmonary arterial hypertension (ICPPS; also known as small patella syndrome), pulmonary arterial hypertension (PAH), and lethal lung developmental disorders. The variability of penetrance and expressivity of *TBX4* variants remains a prominent challenge in understanding their genotype–phenotype correlations. We investigated a five‐generation family with 12 affected individuals presenting with isolated ICPPS, lung‐related manifestations with features of ICPPS, and other milder abnormalities. Whole‐genome sequencing was used to identify the causative and putative modifying variants. Wefound a complex genomic rearrangement (CGR) involving the *TBX4* promoter and its 5 ^′^ untranslated region that segregated in the family. This CGR consists of an ~38 bp insertion, an ~24 bp deletion, an ~2.4 kb deletion, and an ~235 bp inversion. Computational analyses in the proband′s mother with pulmonary and skeletal manifestations revealed 27 candidate modifying noncoding SNVs in the *TBX4* lung‐specific super‐enhancer and 45 SNVs within its topologically associating domain (TAD), including seven variants within the *TBX4* promoter. To explain the variable expressivity of this CGR, we propose that one or more of the variants within the *TBX4* lung‐specific super‐enhancer or TAD may act in *trans* with the pathogenic CGR, modulating *TBX4* expression from the intact allele.

## 1. Introduction

The *TBX4* gene on chromosome 17q23.2 exhibits broad spatial and temporal expression patterns and participates in major developmental signaling pathways in a tissue‐specific manner [1].

In the lung, *TBX4* is expressed in the mesenchyme of the developing buds where it interacts with key signaling molecules, including FGF10, WNT2, and BMP to regulate branching morphogenesis, alveologenesis, and pulmonary vasculature development [2]. It is also involved in limb development [3].

Heterozygous variants in *TBX4* have been associated with a continuum of clinical presentations, ranging from ischiocoxopodopatellar syndrome with or without pulmonary arterial hypertension (PAH) (ICPSS; also known as small patella syndrome; SPS; MIM# 147891) [4], PAH [5], and lethal lung developmental disorders (LLDD) [6]. Homozygous or compound heterozygous variants in *TBX4* are associated with posterior amelia, with pelvic and pulmonary hypoplasia syndrome (MIM# 601360) [7].

Observed incomplete penetrance and variable expressivity imply the contribution of modifying alleles and/or environmental factors [6, 8]. The severity and age of onset of pulmonary manifestations can vary widely, with some individuals exhibiting neonatal respiratory failure, whereas others developing adult‐onset PAH with or without SPS [1, 9]. Genotype–phenotype correlations suggest that loss‐of‐function variants tend to produce more severe pulmonary and skeletal manifestations [1], whereas missense variants, especially those with gain of function, may lead to milder or isolated features [9], including SPS or idiopathic PAH. However, missense variants, including those with a dominant‐negative effect, or loss‐of‐function have been found in patients with LLDDs, including acinar dysplasia (AcDys), congenital alveolar dysplasia (CAD), and other unspecified pulmonary hypoplasia associated with intractable respiratory failure and severe PAH [1, 5].

Here, we ascertained a five‐generation family carrying a complex genomic rearrangement (CGR) involving the *TBX4* promoter region and 5′ noncoding portion of the *TBX4* canonical isoform, incuding noncoding exon 1 that segregates with variable clinical manifestations among multiple family members.

## 2. Materials and Methods

### 2.1. Ethics Statement

All the affected individuals were enrolled under the protocol approved by the Institutional Review Board (IRB) for Human Subject Research at Baylor College of Medicine (H‐8712). All subjects or their legal guardians gave written consent for participation in the study and publication of genomic and clinical information.

### 2.2. Clinical Description

**Figure 1:**
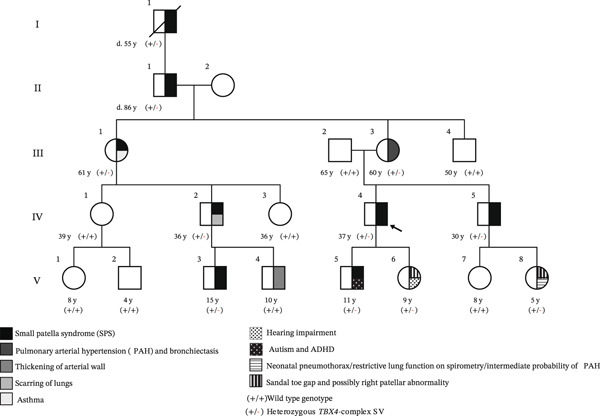
Pedigree of the five‐generation family with a disease‐segregating complex genomic rearrangement (CGR) involving the promoter region and exon 1 of the canonical isoform of the *TBX4* gene. The proband (IV.4), his sibling (IV.5), and his son (V.5) have small patella syndrome (SPS), whereas his daughter (V.6) and niece (V.8) have sandal gaps. V.6 also has hearing impairment and V.8 has history of neonatal pneumothorax and restrictive lung function on spirometry with a probability of pulmonary hypertension (PAH). Proband′s mother (III.3) with PAH and bronchiectasis carries the same variant. Her sibling (III.1) has SPS and asthma, and her son (IV.2) has SPS and scarring of lungs. One of her grandsons (V.3) has SPS, whereas the other grandson (V.4) has thickening of pulmonary arterial wall; however, no CGR was found in him.

We report on a five‐generation family with 12 affected individuals (Figure 1). The 37‐year‐old male proband (IV.4), his sibling (IV.5), and son (V.5) presented with SPS detected on radiographic evaluation. V.5 also has sandal toe gap and was diagnosed with autism and attention deficit hyperactivity disorder. His forced expiratory volume in 1 second (FEV1) was 119% (reference range: 80%–120%) and his forced vital capacity (FVC) was 108% (80%–120%), indicative of normal lung function. The proband′s daughter (V.6) also has sandal toe gap and possibly right patellar abnormality. She was also noted to have poor hearing on screening test. Her FEV1 was 93% and FVC was 85%.

**Figure 2:**
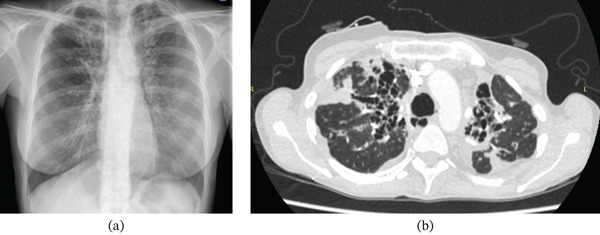
Lung manifestations in patient III.3. Chest x‐ray of III.3 (at the age of 59) demonstrates marked bronchiectasis, with increased hyperinflation and more extensive upper lobe involvement. (a) Diffuse interstitial shadowing is seen in the left mid zone (LMZ) and both lower zones, suggestive of infection, fibrosis, and bronchiectasis and diverticula are seen along the trachea, major airways, and extending to the peripheral bronchi, consistent with Munier‐Kuhn Type III. (b) Additional findings include mucus plugging and parenchymal infection.

Proband′s mother (III.3) has SPS and PAH with bronchiectasis. On chest x‐ray at the age of 59, marked bronchiectasis with increased hyperinflation, extensive upper lobe involvement, and changes in the left mid zone and both lower zones, including interstitial shadowing, suggestive of bronchiectasis, fibrosis, and possible fluid accumulation within the cavity in the left lung with the sagittal tracheal diameter of 26 mm were noted (Figure 2a). Computed tomography (CT) of her lungs revealed a 23‐mm sagittal tracheal diameter, a 12‐mm left main bronchus diameter, and a 14.7‐mm right main bronchus diameter with the presence of diverticula at the level of the trachea, suggestive of Mounier‐Kuhn syndrome, also called tracheobronchomegaly, characterized by abnormal dilatation of the trachea and main bronchi (Figure 2b). Proband′s maternal aunt (III.1) has SPS and asthma; her son (IV.2) has SPS and scarring of lungs and one of her grandsons V.3 has SPS, whereas the other grandson (V.4) has thickening of pulmonary arterial wall. Proband′s niece (V.8) has a history of neonatal pneumothorax, sandal toe gap, and intermediate probability of PAH on noninvasive assessment.

### 2.3. Molecular Analysis

DNA was extracted from peripheral blood using Puregene Blood Core Kit (Qiagen, Germantown, Maryland).

Short read whole‐genome sequencing (WGS) was done in the proband (IV.4), his similarly affected sibling (IV.5), and their mother (III.3) and father (III.2) using a TruSeq Nano DNA HT Library Prep Kit and the NovaSeq X Plus (Illumina, San Diego, California) with mean coverage depth of 30x at Novogene Corporation Inc. (Sacramento, California). Manual inspection and visualization of the *TBX4* locus was performed using Integrative Genomic Viewer (IGV) and Sequencher v.5.4.6 (Gene Codes Co., Ann Arbor, Michigan). Multiallelic SNVs and indels were decomposed using the BCFtools norm, and annotation was performed with the Ensembl Variant Effect Predictor (VEP, release 114). VEP was configured to report a single transcript consequence per variant, selected according to the default Ensembl ranking system, which prioritized Matched Annotation from NCBI (National Center for Biotechnology Information) and EMBL‐EBI (MANE) Select, MANE Plus Clinical, canonical, and APPRIS (annotation of principal and alternative splice isoforms) transcripts, followed by those with strong transcript support levels, protein‐coding biotype, consensus coding sequence (CCDS) membership, greater predicted consequence severity, and transcript length.

Long‐read whole‐genome sequencing (LR WGS) was performed using PacBio HiFi technology with 30x coverage at Novogene Corporation Inc. (Sacramento, California). Haplotype phasing was done using SAMtools.

The canonical *TBX4* (NM_001321120.2) isoform was used as a reference. Breakpoint junctions were verified using Sanger sequencing. We analyzed and catalogued the variants within the *TBX4* lung‐specific super‐enhancer [chr17: 61,201,663‐61,384,701] [6], promoter(s), and TAD [chr17: 61,348,835‐61,575,000] (GRCh38/hg38) in the WGS data from the proband (IV.4), his brother (IV.5), and mother (III.3), all carriers of CGR and presenting with skeletal and/or pulmonary phenotypes, as well as in the phenotypically unaffected proband′s father (III.2). We also annotated individual variants with their corresponding dbSNP identifiers (if available), allele frequencies (gnomAD v4.1, ALFA, and *All of Us* databases), ClinVar ID, PubMed ID, and in silico predictions (CADD, REVEL, SIFT, and PolyPhen scores).

### 2.4. Codex‐Assisted Variant Prioritization

We employed an OpenAI agent (Codex) to integrate multiple complementary variant‐level metrics, including allele frequency, prior literature evidence, CADD scores, Enformer predictions, AlphaGenome annotations, and related contextual evidence. Codex generated a heuristic assessment for variant prioritization and assigned priority tiers based on the combined evidence.

### 2.5. Reporter Assay

We PCR‐amplified ~2 kb genomic region from patient IV.5′s wild‐type and rearranged copies of chr17, extending from the *TBX4* gene upstream to genomic position 61,450,345 (GRCh38/hg38), and have cloned it into the *Bgl*II and *Hin*dIII sites in the multiple cloning site of the promoter‐less pGL4.10‐based *luc2* expression vector. For transfection, human fetal lung fibroblasts, IMR‐90 (ATCC, Manassas, Virginia), were cultured in FBS‐supplemented EMEM medium (ATCC) on 12‐well plates. The cells were transfected with 1 *μ*g/well of tested DNA and 0.1 *μ*g pGL4.75 (constitutively expressing *Rluc*) using Lipofectamine 3000 (Invitrogen, Waltham, Massachusetts) (4 *μ*l/well). RNA was isolated 48 h after transfection and converted to cDNA using SuperScript III kit (Invitrogen). The expression of *luc2* or *Rluc* was determined by quantifying their cDNA by qPCR. Custom‐designed TaqMan primers and probes (*luc2* assay AP7DRTC and *Rluc* assay AP47W76) were obtained from Applied Biosystems (Waltham, Massachusetts). For relative quantification of cDNA/transcripts, the comparative C_T_ method was used. *Luc2* levels were normalized to those of *Rluc*.

## 3. Results

### 3.1. Molecular Findings

We have identified a heterozygous CGR encompassing the putative *TBX4* promoter, the noncoding exon 1, and portion of intron 1 of the longer isoform of *TBX4* (NM_001321120.2), consisting of an ~38 bp insertion (chr17: 61,451,389‐61,451,425), an ~24 bp deletion (chr17:61,451,654‐61,451,677), an ~2.4 kb deletion (chr17: 61,451,725‐61,454,136), and an ~235 bp inversion (chr17: 61,451,770‐61,452,005) (GRCh38/hg38) (Figures S1, S2, S3, and S4). This rearrangement segregates with the disease within the family.

**Figure 3:**
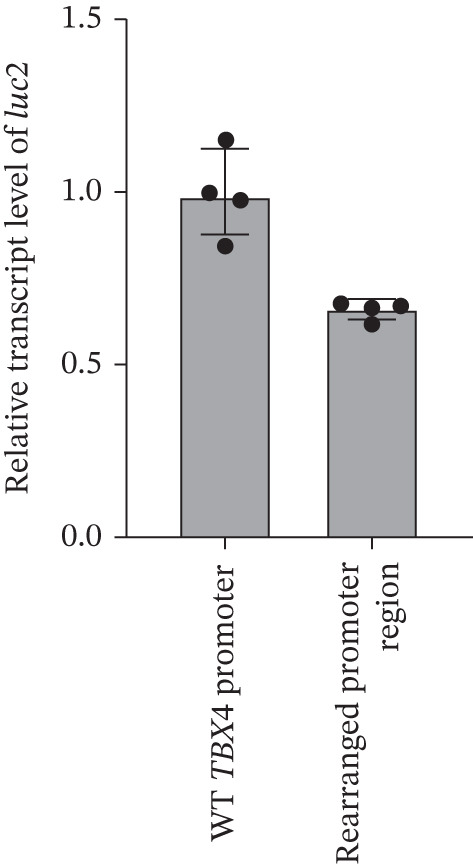
Decreased *TBX4* promoter activity due to complex genomic rearrangement. Luciferase reporter assay was used to assess promoter activity of the wild‐type and rearranged *TBX4* promoter regions. The rearranged promoter exhibited significantly reduced transcriptional activity compared with the wild‐type construct. Data are presented as mean ± SD from independent experiments. Statistical significance was determined using an unpaired two‐tailed Student′s *t*‐test (*p* < 0.01).

The reporter assay demonstrated that deletion of the *TBX4* core promoter element (ENCODE4 cCRE: EH38E3235375) and associated rearrangements of its flanking sequences resulted in a 34% reduction (*p* < 0.01) in the basal activity of the promoter region (Figure 3). Notably, the actual reduction in *TBX4* expression was likely much greater due to the loss of interactions of the deleted promoter with the enhancer.

Overall, we identified 27 noncoding SNVs and small indels within the lung‐specific super‐enhancer of *TBX4* in the proband′s mother (III.3) that are absent in the proband (IV.4), his brother (IV.5), and father (III.2). The minor allele frequencies (MAFs) of these 27 variants in gnomAD v4.1 range from 0.00004 to 0.42 (Table S1). (Table S1). Most variants demonstrated low‐to‐moderate CADD PHRED scores, with only a few exceeding a PHRED score of 10, whereas Enformer predicted generally modest changes in regulatory sequence activity (SAD/SAR). Out of these, 22 variants map to the transcription factor binding sites or regions marked by active or poised chromatin (Figure S5 , Table S1). Using the ENCODE ChIP‐seq Transcription Factor Cluster data (UCSC Genome Browser), we have found that 22 out of 27 SNPs foundwithin the *TBX4* super‐enhancer in the proband’s mother map to the TF‐binding hotspots, harboring transcription factor binding sites, and fall within the chromatin domains marked as active or poised (Table S1). They also map to ATAC‐seq peaks identified in IMR‐90 human fetal lung fibroblast cell line and tohighly conserved genomic regions (Figure S5). In the promoter region of *TBX4*, there are no unique variants in the proband′s mother (Table S2). Additionally, we also identified in the proband′s mother 45 SNVs and small indels within the TAD of *TBX4* with their MAFs in gnomAD v4.1 ranging from 0.0001314 to 0.42 (Table S3).

**Figure 4:**
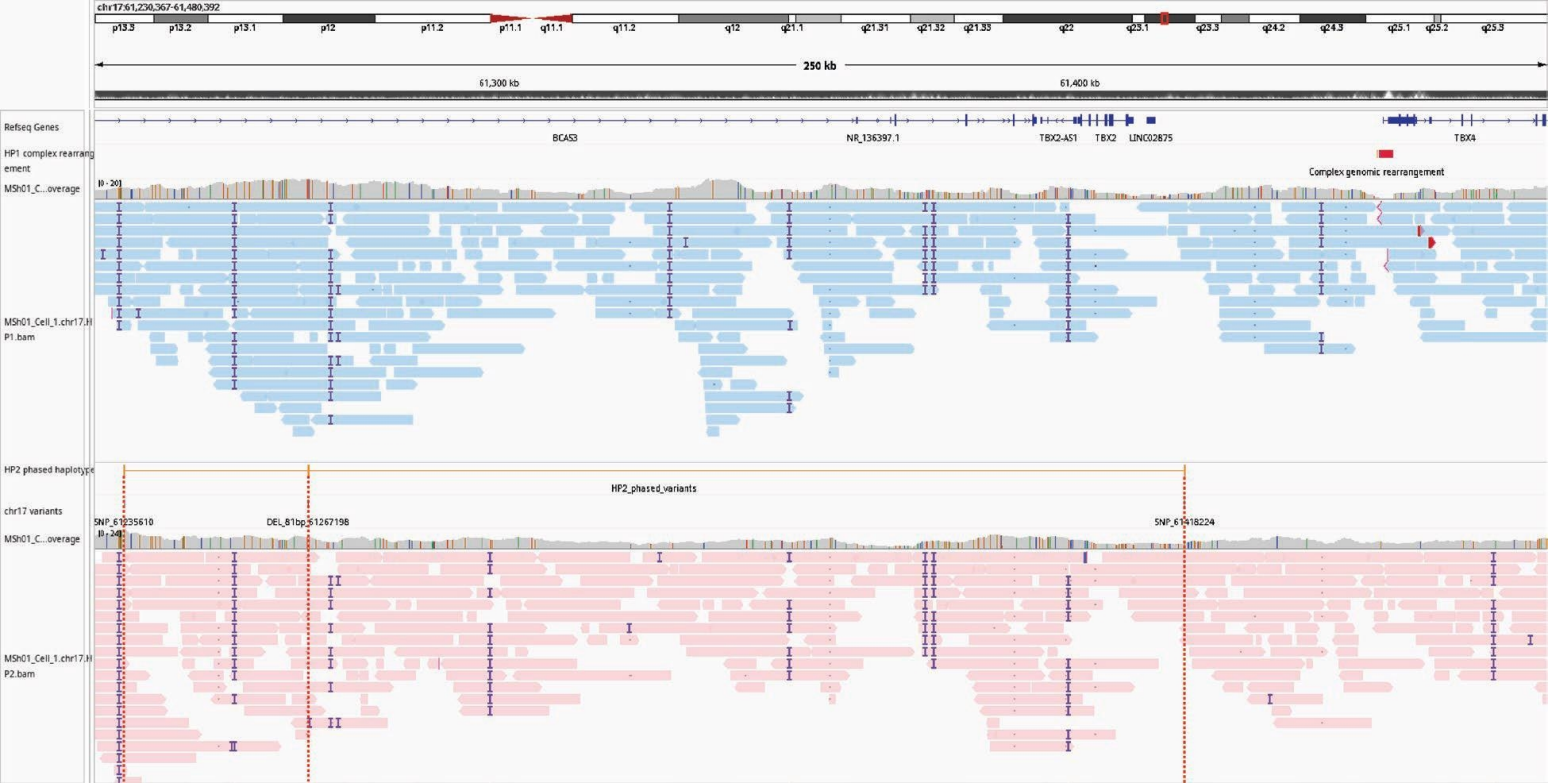
IGV visualization of haplotype‐phased long reads. Note that three candidate variants, rs997855798, rs118168361, and rs1568707257 (bottom, grandmaternal allele), are located in *trans* to the described CGR (top, grandpaternal allele).

Using the Codex framework, we have identified and prioritized three candidate modifier variants including rs997855798, rs118168361, and rs1568707257 found only in III.3. Using pedigree segregation and LR WGS analyses, we have determined that they are located in *trans* to the *TBX4* CGR (Figure 4, Table S4).

## 4. Discussion

*TBX4* exhibits substantial transcript complexity, with two major isoforms arising from the alternative promoters [12]. The canonical transcript NM_001321120.2 comprises nine exons and encodes a 546‐amino acid protein, and the second isoform NM_018488.3 consists of eight exons and encodes a 545‐amino acid protein. The conserved T‐box DNA‐binding domain, located proximally at the N‐terminus, encompasses residues 71–251 and is encoded by a part of exon 3 through a portion of exon 7 [13]. The described CGR harbors the promoter, noncoding exon 1, and a portion of intron 1 of the canonical *TBX4* isoform NM_001321120.2, implying that pathogenicity is more likely due to transcriptional dysregulation rather than disruption of the protein‐coding sequence.

Previous studies on other T‐box family members, including *TBX3*, *TBX5*, and *TBX20*, have shown isoform‐ and tissue‐specific expression patterns, indicating that alternative splicing contributes to tissue‐specific functional outcomes [14]. However, similar mechanisms have not been described for *TBX4* (Figure S6). The pulmonary manifestations seen in four individuals in this family may be influenced by putative modifiers that specifically impact *TBX4* expression in lung tissue. However, current evidence is insufficient to conclusively establish tissue‐specific expression differences between these isoforms or to define their functional relevance, and further studies are warranted to validate this hypothesis.

Studies of families with rare LLDDs have revealed a few heterozygous coding variants involving *TBX4* inherited from asymptomatic, mildly affected parents, or with variable phenotypic manifestations, suggesting that they alone may be insufficient to cause the specific phenotypes and indicated the potential role of noncoding regulatory variants *e.g.,* hypomorphic alleles. The authors proposed a complex compound inheritance model with a combination of coding and in *trans* noncoding variants that may explain variable expressivity of *TBX4*‐related disorders [6, 15].

In this study, in the proband′s mother we have identified and catalogued 27 noncoding variants within the lung‐specific super‐enhancer and 45 variants within the TAD of *TBX4,* some or many of which may contribute to the observed variable phenotypic expression. Importantly, two SNPs, rs35383405 (chr17_61342791‐G‐T) [6, 11] and rs929472 (chr17‐61329990‐C‐T) [10] were previously proposed as putative hypomorphic alleles associated with *TBX4*‐associated LLDDs, making them good candidates for phenotypic modifiers in the proband′s mother. Twenty‐two SNPs in the predicted lung‐specific super‐enhancer region overlap multiple transcription factor‐binding sites, suggesting their potential role in modifying interactions between transcription factors and the enhancer. Their location within chromatin marked as active or poised, IMR‐90 ATAC‐seq peaks, and within the evolutionary conserved region indicate that they likely reside in the accessible and potentially functional regulatory domains, thus potentially affecting the interaction of the transcription factors with the enhancer. Further experimental studies are needed to determine whether any of these variants exert modification or regulatory activity. Three variants rs997855798, rs118168361 and rs1568707257, identified using Codex, LR WGS, and family segregation analyses map in *trans* to the described CGR (Figure 4, Table S4). Although these findings do not establish a causal modifier effect, they provide additional evidence that thesevariants may represent potential candidate modifiers contributing to phenotypic variability noted in the family.

We have also noted three SNPs in the promoter region of *TBX4*, rs4141079, rs4968564, and rs11867179 shared among all four individuals (III.3, IV.4, IV.5, and III.2) that were previously reported as associated with hip shape variance [16], nonsyndromic clubfoot [17] and breast cancer risk [18], respectively, whereas 45 SNVs/indels found within the TAD of *TBX4* were not previously associated with any phenotypes.

The reporter assay demonstrated that deletion of the *TBX4* core promoter element (ENCODE cCRE: EH38E3235375), together with rearrangement of its flanking sequences, resulted in a significant 34% reduction in basal promoter activity. However, this reduction is likely to underestimate the overall impact of the variant on *TBX4* expression as in addition to the promoter deletion leading to impaired transcription initiation, this genomic rearrangement could also likely eliminate functional interactions with distal enhancers that contribute to *TBX4* regulation.

Genomic structural impediments can trigger replication stress, cause DNA replication fork stalling or collapsing, predisposing to template switching during lagging‐strand synthesis as the cell attempts to restart DNA synthesis [19]. For the described CGR, we propose a multiple template switch fork stalling and template switching/microhomology‐mediated break‐induced replication (FoSTeS/MMBIR) mechanism of formation [20]. The nascent strand may have dissociated and reannealed to a nearby microhomologous sequence (2–10 bp), causing the polymerase to skip a 24 bp segment and generate the first small deletion. A second switch likely redirected synthesis to a nearby inverted complement, producing the 235 bp inversion within the promoter. A third switch may have occurred when the strand failed to return to the downstream template and instead annealed further away, forming the 2411 bp deletion. Finally, the 34–38 bp inserted fragment was likely copied from a nearby locus during a transient mis‐annealing event before the strand returned to the main template.

In summary, although the identified noncoding variants represent putative modifiers, the lack of DNA from additional affected relatives precluded genomic burden analysis. Thus, their contribution to the observed phenotypic variability and validation in additional families with further functional studies remains a limitation of our study. Functional validation will be essential to assess their effects, including promoter‐reporter and expression studies, to determine whether these regulatory changes alter *TBX4* isoform expression and contribute to the observed phenotypic variability.

## Author Contributions

S.A.P. has contributed to data analysis, writing the manuscript and preparing the tables and figures; H.C.J. contributed in preparing the figures; T.G. contributed in data generation and analysis; Pr.S. performed the reporter assay and contributed to data analysis; J.A.K. contributed to data analysis and manuscript reviewing and overall supervision; F.P.E., R.E.T., M.J.P., C.H., and S.M. have contributed to patient evaluation, patient referral and radiodiagnosis; Pa.S. has contributed to patient recruitment, analysis, interpretation of the genomic testing, planning and conceptualizing the manuscript and overall supervision. S.A.P., H.C.J, and Pr.S. are equal contributors.

## Funding

This study was supported by the National Institutes of Health (1R01HL165301‐01A1) for Pa.S. and the National Science Centre in Poland, 2019/35/D/NZ5/02896 for J.A.K.

## Conflicts of Interest

The authors declare no conflicts of interest.

## Supporting information


**Supporting Information** Additional supporting information can be found online in the Supporting Information section. Table S1: Candidate SNVs and small indels identified within the *TBX4* lung‐specific super‐enhancer. Table S2: Candidate SNVs and small indels identified within the *TBX4* promoter. Table S3: Candidate SNVs and small indels identified within the *TBX4* TAD. Table S4: Three variants, rs997855798, rs118168361, and rs1568707257, identified using Codex, family pedigree segregation, and long read WGS analyses to map in *trans* to the described complex genomic rearrangement (CGR). Figure S1: Integrated genomic viewer (IGV) visualization of the described complex genomic rearrangement (CGR) involving the *TBX4* promoter region. Figure S2: Schematic representation of chromosome 17q23.2 region encompassing CGR involving the putative regulatory promoter region, exon 1, and part of intron 1‐2 of *TBX4*. Figure S3: IGV visualization of CGR with the family pedigree. Figure S4: Proposed mechanism of CGR formation. Figure S5: Genomic context of the putative regulatory SNPs within the *TBX4* enhancer. Figure S6: Transcript‐level expression of *TBX4* across human tissues based on the GTEx data.

## Data Availability

The data that support the findings of this study are available from the corresponding authors upon reasonable request.
