# Supplementary material for: Complex Genomic Rearrangement Involving the TBX4 Promoter Manifesting With Variable Expressivity in a Five‐Generation Family
Source: Hum Mutat. 2026 Aug 3;2026:7278477. doi: 10.1155/humu/7278477 (PMC13430282; doi:10.1155/humu/7278477)
Supplement: Supplementary file 1 — Supporting Information Additional supporting information can be found online in the Supporting Information section. Table S1: Candidate SNVs and small indels identified within the TBX4 lung‐specific super‐enhancer. Table S2: Candidate SNVs and small indels identified within the TBX4 promoter. Table S3: Candidate SNVs and small indels identified within the TBX4 TAD. Table S4: Three variants, rs997855798, rs118168361, and rs1568707257, identified using Codex, family pedigree segregation, and long read WGS analyses to map in trans to the described complex genomic rearrangement (CGR). Figure S1: Integrated genomic viewer (IGV) visualization of the described complex genomic rearrangement (CGR) involving the TBX4 promoter region. Figure S2: Schematic representation of chromosome 17q23.2 region encompassing CGR involving the putative regulatory promoter region, exon 1, and part of intron 1‐2 of TBX4. Figure S3: IGV visualization of CGR with the family pedigree. Figure S4: Proposed mechanism of CGR formation. Figure S5: Genomic context of the putative regulatory SNPs within the TBX4 enhancer. Figure S6: Transcript‐level expression of TBX4 across human tissues based on the GTEx data. [file HUMU-2026-7278477-s001.zip › Supplement_revised.docx]

**Supplemental Material**

**Complex genomic rearrangement involving the *TBX4* promoter manifesting with variable expressivity in a five-generation family**

Shruti A. Pande^1^, Hiuling Chan Joiner^1^, Tomasz Gambin^2^, Przemyslaw Szafranski^1^, Frank P. Edenborough^3^, Rachael E. Thompson^3^, Michael J. Parker^4^, Carrie Hammond^4^, Shahin Moledina^5^, Justyna A. Karolak^6^ and Pawel Stankiewicz^1^

**Table S1.** Candidate SNVs and small indels identified within the *TBX4* lung-specific super-enhancer.

**Table S2.** Candidate SNVs and small indels identified within the *TBX4* promoter.

**Table S3.** Candidate SNVs and small indels identified within the *TBX4* topologically associating domain (TAD).

**Table S4.** Prioritized candidate modifier variants

**Figure S1.**

**
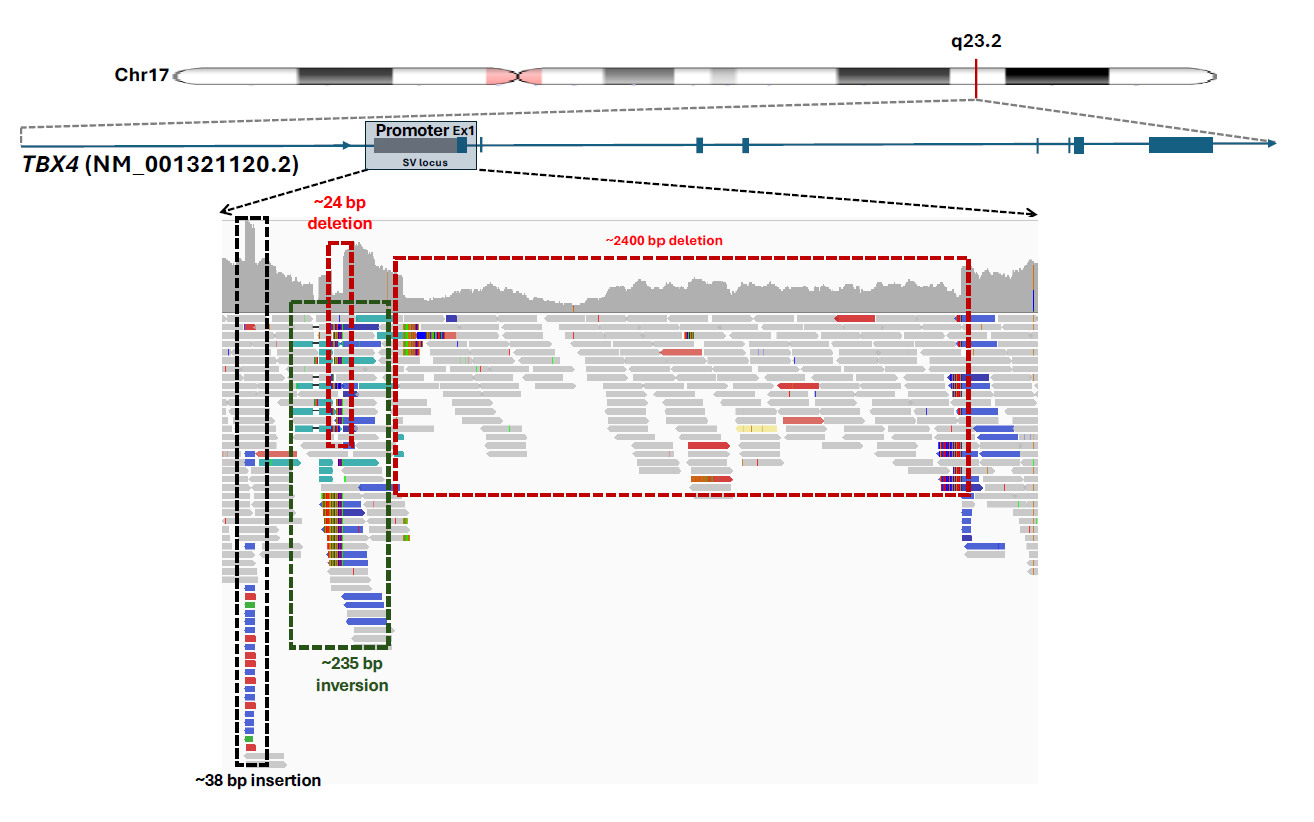
**

**Figure S1. Integrated genomic viewer (IGV) visualization of the described complex genomic rearrangement (CGR) involving the *TBX4* promoter region.**

Ideogram of chromosome 17 showing the location of the *TBX4* gene at 17q23.2. The region is zoomed in to illustrate the complex structural variant (SV) identified in the promoter and exon 1 of canonical *TBX4* isoform*.* IGV visualization of aligned sequencing reads (BAM file) highlights the key rearrangement components: an ~38 bp insertion (black dotted box), a ~24 bp deletion (red dotted box), a ~235 bp inversion (green dotted box), and a ~2400 bp deletion (red dotted box).

**Figure S2.**

**
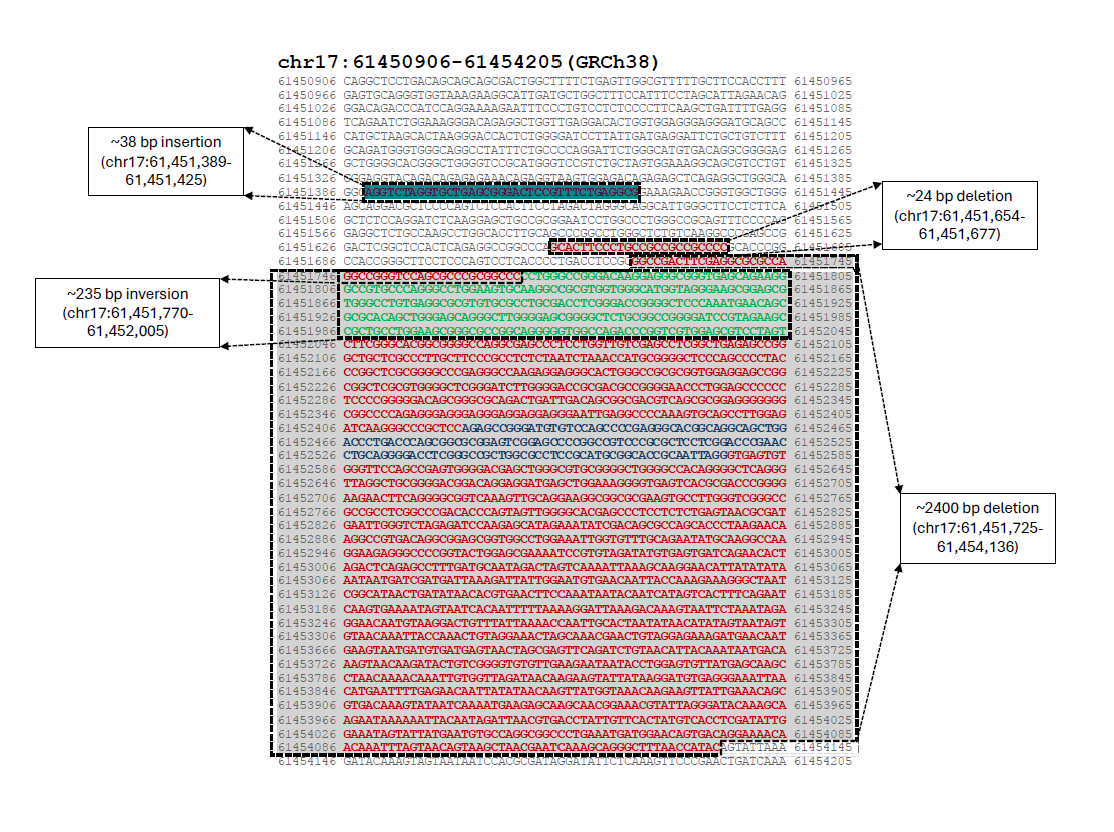
**

**Figure S2. Schematic representation of chromosome 17q23.2 region encompassing the CGR involving the putative regulatory promoter region, exon 1, and part of intron 1-2 of *TBX4*.** Color coding denotes the following features: the violet font highlighted in blue indicates a ~38 bp insertion (chr17:61,451,389-61,451,425), the grey highlight indicates the deleted region; within this, the red highlight represents a ~24 bp deletion (chr17:61,451,654-61,451,677), and the larger ~2400 bp deletion (chr17:61,451,725-61,454,136) is also within the grey-highlighted region, the green font within the grey region marks an inversion of ~235 bp (chr17:61,451,770-61,452,005) while the blue font within the grey highlighted region marks the exon 1 of *TBX4*.

**Figure S3.**

**
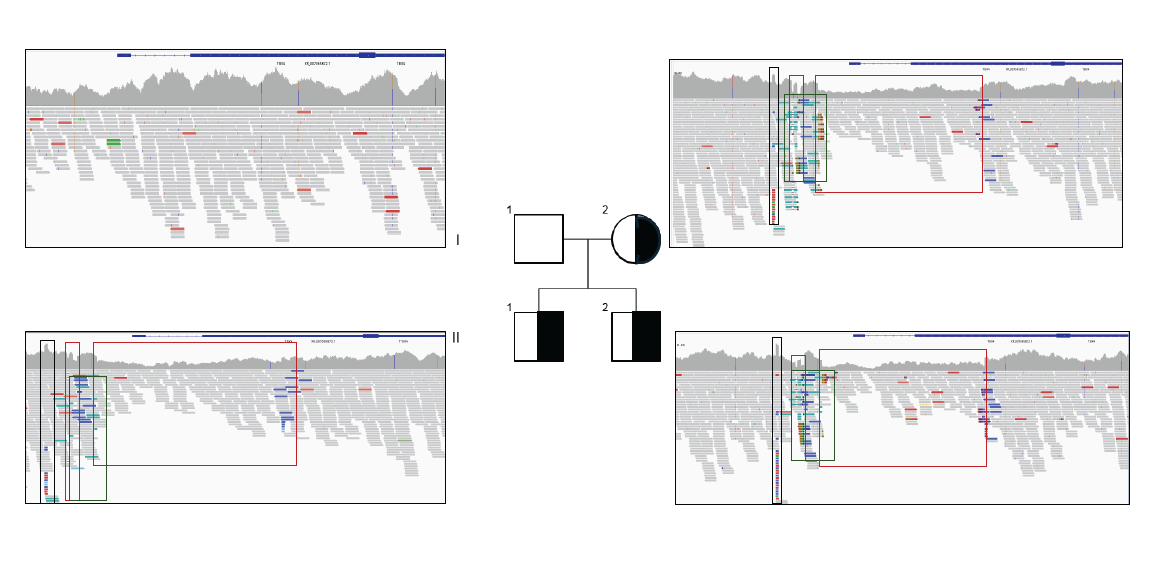
**

**Figure S3. IGV visualization of CGR with the family pedigree.**

Visualization of BAM files in IGV for the proband (II.1), his affected brother (II.2) and their parents (I.1 and I.2). I.1 carries the wild-type genotype, while a complex structural variant (SV) involving the promoter region and exon 1 of *gene TBX4* is observed in the proband, the affected sibling, and their mother. Color coding represents the respective SVs: red dotted boxes indicate the smaller deletion of ~24 bp deletion (chr17:61,451,654-61,451,677), and larger ~2400 bp deletion (chr17:61,451,725-61,454,136), the black dotted box indicates the ~38 bp insertion (chr17:61,451,389-61,451,425), and the green dotted box indicates the inversion of ~235 bp (chr17:61,451,770-61,452,005).

**Figure S4.**

**
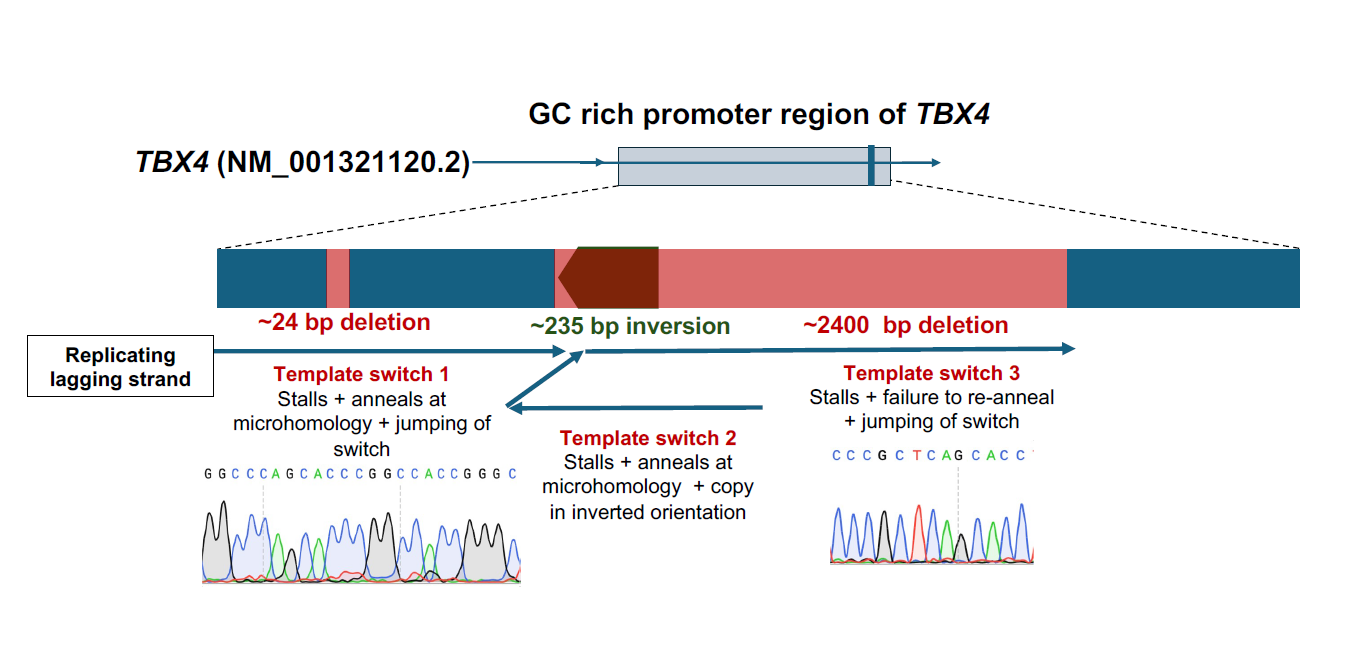
**

**Figure S4. Proposed mechanism of CGR formation.**

The panel depicts the proposed replication-based mutagenesis mechanism underlying this rearrangement. The GC-rich promoter region of *TBX4* likely predisposes to replication fork stalling or fork stress, initiating a first template switch. Using short microhomologies, the nascent strand undergoes microhomology-mediated annealing, skipping over the 24 bp segment and generating the first deletion. A second template switch occurs when the strand anneals to an inverted template orientation, resulting in the ~ 235 bp inversion. A third switch re-anneals further downstream, leading to loss of ~ 2411 bp. Finally, a short 34–38 bp fragment is transiently copied from a nearby genomic locus, forming the observed templated insertion.

**Figure S5.**


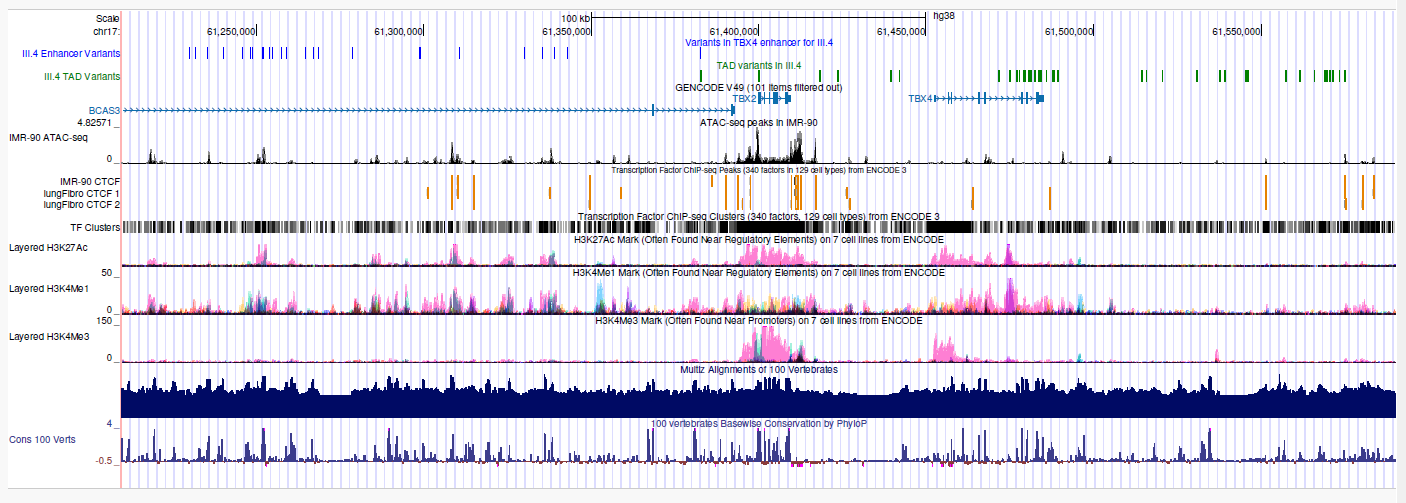


**Figure S5. Genomic context of the putative regulatory SNPs within the *TBX4* enhancer.**

All the 27 SNPs within the putative lung-specific enhancer of *TBX4* are represented by blue bars in the first row. Most of these are positioned within a transcription factor–binding hotspots in the lung-specific super-enhancer region of *TBX4*. These SNPs overlap the predicted *TBX4* lung-specific super-enhancer and promoters and coincide with the multiple transcription factor ChIP-seq peaks and chromatin regions marked as active or poised. They also align with the IMR-90 ATAC-seq peaks and highly conserved genomic sequences. Previously reported rs35383405 and rs929472 are shown in red.^6,14,15^

**Figure S6.

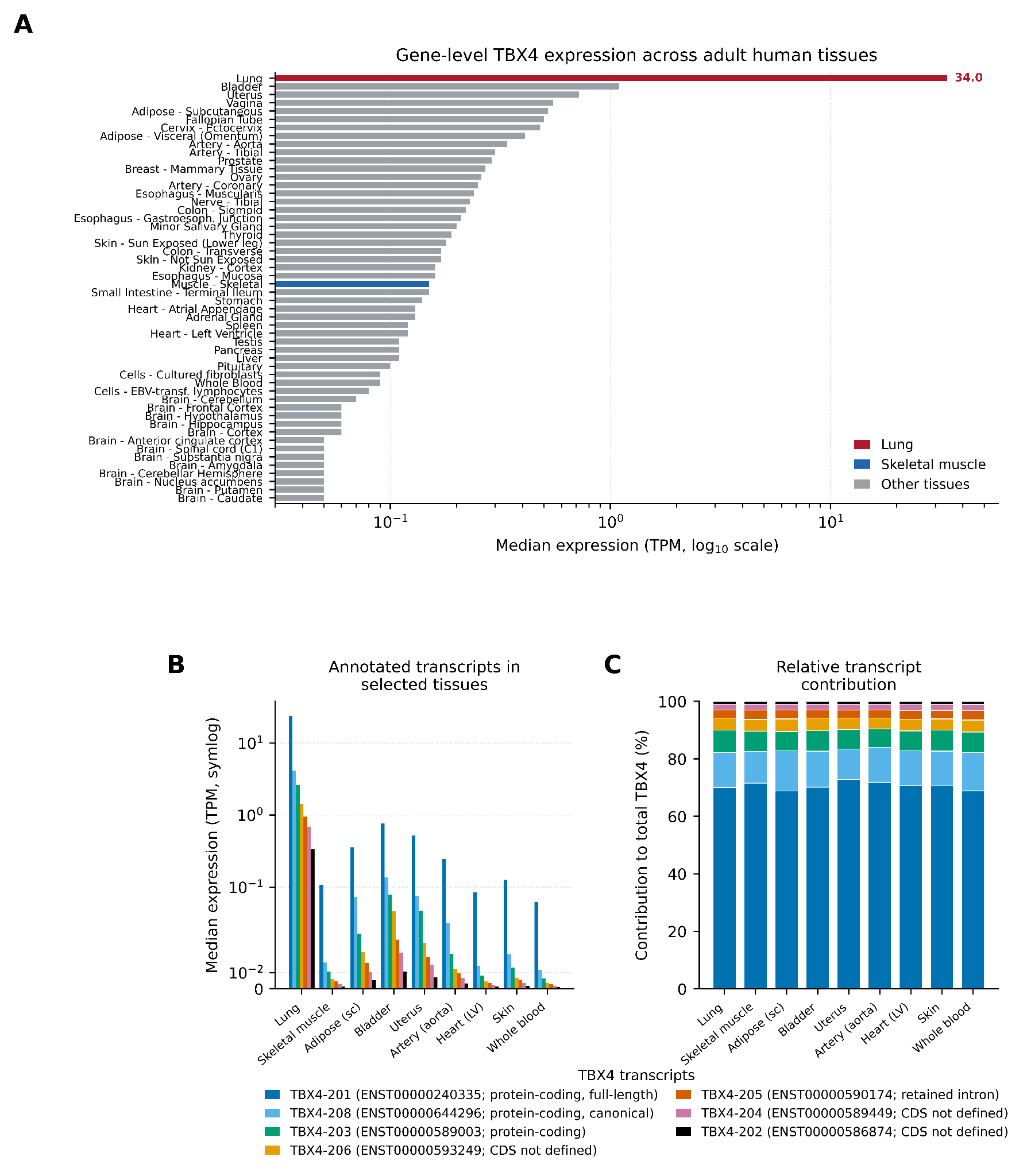
**

**Figure S6. Transcript-level expression of *TBX4* across human tissues based on the GTEx data*.*** (A) Gene-level *TBX4* expression across human tissues shown as median transcripts per million (TPM) on a log₁₀ scale and ranked from highest to lowest. Lung (red) shows markedly higher *TBX4* expression than all other tissues; skeletal muscle (blue) is highlighted for reference and lies near the low end of the distribution. (B) Expression of the individual annotated *TBX4* transcripts (Ensembl/GENCODE identifiers) in a panel of selected tissues spanning lung, skeletal muscle, and a representative spread of other tissue types; median TPM is plotted on a symmetric-log axis to accommodate the wide dynamic range. (C) Relative contribution of each transcript to total *TBX4* expression in the same tissues, expressed as a percentage of summed transcript TPM. The full-length protein-coding isoform TBX4-201 (ENST00000240335) predominates in every tissue examined, with the remaining transcripts contributing minor and broadly consistent fractions.
